# Development and application of a modified dynamic time warping algorithm (DTW-S) to analyses of primate brain expression time series

**DOI:** 10.1186/1471-2105-12-347

**Published:** 2011-08-18

**Authors:** Yuan Yuan, Yi-Ping Phoebe Chen, Shengyu Ni, Augix Guohua Xu, Lin Tang, Martin Vingron, Mehmet Somel, Philipp Khaitovich

**Affiliations:** 1 Key Laboratory for Computational Biology, CAS-MPG Partner Institute for Computational Biology, Shanghai Institutes for Biological Sciences, Chinese Academy of Sciences, 320 Yue Yang Road, Shanghai 200031, China; 2 Department of Computer Science and Computer Engineering, La Trobe University, Melbourne, VIC 3086, Australia; 3 Max Planck Institute for Evolutionary Anthropology, Deutscher Platz 6, D-04103 Leipzig, Germany; 4 Max Planck Institute for Molecular Genetics, Ihnestrasse 63-73, D-14195 Berlin, Germany

## Abstract

**Background:**

Comparing biological time series data across different conditions, or different specimens, is a common but still challenging task. Algorithms aligning two time series represent a valuable tool for such comparisons. While many powerful computation tools for time series alignment have been developed, they do not provide significance estimates for time shift measurements.

**Results:**

Here, we present an extended version of the original DTW algorithm that allows us to determine the significance of time shift estimates in time series alignments, the DTW-Significance (DTW-S) algorithm. The DTW-S combines important properties of the original algorithm and other published time series alignment tools: DTW-S calculates the optimal alignment for each time point of each gene, it uses interpolated time points for time shift estimation, and it does not require alignment of the time-series end points. As a new feature, we implement a simulation procedure based on parameters estimated from real time series data, on a series-by-series basis, allowing us to determine the false positive rate (FPR) and the significance of the estimated time shift values. We assess the performance of our method using simulation data and real expression time series from two published primate brain expression datasets. Our results show that this method can provide accurate and robust time shift estimates for each time point on a gene-by-gene basis. Using these estimates, we are able to uncover novel features of the biological processes underlying human brain development and maturation.

**Conclusions:**

The DTW-S provides a convenient tool for calculating accurate and robust time shift estimates at each time point for each gene, based on time series data. The estimates can be used to uncover novel biological features of the system being studied. The DTW-S is freely available as an R package *TimeShift *at http://www.picb.ac.cn/Comparative/data.html.

## Background

Comparing and characterizing temporal changes in gene expression is a routine method to elucidate functional features of biological processes [1,2]. If gene expression levels are measured across time in different individuals, or under different conditions, it may be desirable to compare the timing of gene expression changes between two or more time series. As the number of large-scale time series experiments increases, so does the need for detailed and comprehensive comparisons between them. Such analyses can reveal dynamic features of the system, such as acceleration or delay in temporal expression changes caused by conditional perturbations, or individual or evolutionary differences. One example of such an analysis was the study of developmental gene expression patterns in two insect species: mosquito (*Anopheles *gambiae) and fruit fly (Drosophila melanogaster) [3]. This analysis, based on the temporal alignment of the gene expression profiles between the two species, revealed distinct groups of genes involved in functions known to be specific to one of the species, including delay in mosquito cuticle synthesis, as well as faster maternal-zygotic transition in flies. Other recent applications of temporal alignment algorithms include: retention time alignment of complex LC-MS data in proteomics and metabolomics studies [4,5], and alignment of transcriptome changes in two types of the cardiac hypertrophy [6].

The methods employed to address such temporal alignment problems are commonly based on the dynamic time warping (DTW) alignment method. DTW was originally developed for dealing with speech recognition problems [7,8]. It utilises dynamic programming to find an optimal alignment with respect to a given scoring function. The application of DTW to gene expression data was pioneered by Aach and Church [9] and has been further developed by other groups [10-14].

In this study, we tested whether the temporal alignment of ontogenetic expression profiles can reveal novel biological features of human brain development. To do so, we used published gene expression time series from the human, chimpanzee and rhesus macaque prefrontal cortex [15], as well as human and rhesus macaque cerebellum [15]. Human development is known to proceed more slowly than development of chimpanzees and macaques. This trend is reflected in longer gestation time (humans - 280 days, chimpanzees - 220 days, rhesus macaques - 165 days), later age of sexual maturity (in humans - 13 years and in the wild; in chimpanzees - 8 years and in rhesus macaques - 6 years), and longer maximal lifespan (humans - 100 years, chimpanzees - 60 years, rhesus macaques - 40 years) [16,17]. Further, transcriptome changes during prefrontal cortex development tend to be delayed in humans compared to chimpanzees [15]. On the phenotypic level, delay in human development with respect to the closely related primate species, such as chimpanzees, was long hypothesized to play an important role in evolution of human cognition [18,19]. Thus, investigation of the temporal differences between developmental gene expression profiles in the human, chimpanzee and rhesus macaque brains using DTW-based alignment method might shed new light on the biological processes underlying human cognitive development.

The common features of our algorithm and other alignment methods [11] include: (a) use of interpolated expression values for two aligned time series based on fitted higher order spline curves, and (b) use of one of the time series as a template for aligning the second one, using a resampling procedure based on Euclidian distance minimization. Our alignment approach, in common with other methods, cannot be applied to genes that have substantially different temporal expression behaviour in different time series. In such cases, the difference between two time series is likely to involve factors other than a change in expression timing and, therefore, time shift estimation may be inappropriate.

One of the advantages of our method, compared to the original DTW algorithm, is that it follows the approach first suggested by Smith *et al*. [11] in lifting the requirement to align the ends of the two time series. This is a small but important modification as for many naturally occurring processes the timing of a process' commencement and termination, as well as the timing of corresponding points across different time series, are not known. This improvement to DTW, introduced in our method, is not based on local alignment as in Smith *et al*. [11], nor based on segment alignment as in Smith *et al*. [14], but on a dynamic programming-based alignment procedure adopted during global alignment.

The novel aspects of our algorithm include it's ability to not only estimate the optimal alignment for each time point of each gene, but also determine the reliability of this estimate. Specifically, we implement a multiple simulation procedure based on real data parameters estimated on a gene-by-gene basis. This allows us to assess the significance of estimated time shift values for each time point of each gene. Furthermore, we are able to determine the global significance level for each gene based on false positive rate (FPR) estimates. The significance estimation is based on implementation of our dynamic programming-based algorithm, which is capable of both simulating thousands of expression profiles, and determining simulated time shift values, at fast speed. This is a unique feature of our algorithm compared to other similar alignment methods.

Our method allows the user to select genes with time shift estimates determined to the desired degree of confidence. The time shift profiles that are obtained, can be further used to group genes according to their alignment properties, rather than their original expression profiles. When we applied our method to published gene expression time series of human and chimpanzee prefrontal cortex development, we were able to determine additional biological information by separating genes showing human-chimpanzee expression divergence into distinct groups, based on their time shift properties. Furthermore, when we applied our method to published gene expression time series of human and rhesus macaque prefrontal cortex and cerebellum development, we found that genes expressed in the gray and white matter of the brain show synchronized time shifts between the two species, despite differences in expression profiles between the brain regions. These examples demonstrate the utility of the DTW-S algorithm, which, by implementing the significance level of the gene-by-gene variable time shift estimates, allows us to obtain novel biological insights from time series data.

## Methods

### The DTW-Significance algorithm (DTW-S)

There were three main motivations to advance the existing DTW algorithm: (i) to find the best alignment between two time series, (ii) to estimate the value of time shift between these series at each time point and, (iii) to estimate probability to observe a given time shift at each time point by chance, when actual time shift between two time series is zero.

DTW-S works as follows: we consider two series, *x *and *y*, with the number of time points *m *and *n *respectively: (*x*_1_,...,*x*_m_) and (*y*_1_,...,*y*_n_), where *n *and *m *can be different. Since expression time series tend to be noisy (due to both technical and biological sources) and samples' ages may not be uniform across the age-range (human/primate samples are often collected opportunistically), we use cubic spline interpolation, as implemented in the *smooth. spline *R function, to construct the age-expression trajectory for each gene within a sample group. This is similar to the approach of Smith *et al*. [11]. To avoid overfitting, a generalized cross-validation procedure using a suitable smoothing parameter is implemented in the *smooth. spline *R function [20]. The degrees of freedom parameter for spline curve fitting of the actual biological data was set to four. This parameter provided a good data fit for the actual number of data measurements (39 for humans and 14 for chimpanzees), determined by visual inspection of individual gene expression profiles. We subsequently reconstruct unobserved expression levels at uniformly distributed time points. This, in turn, enables us to estimate the time shift between two time series with greater precision. We denote the interpolated time series by X and Y: (*X*_1_,..., *X*_M_) and (*Y*_1_,..., *Y*_N_).

Our dynamic programming-based algorithm aims to find an optimal alignment between two time series (X and Y with number of time points *M *and *N *respectively, *M*>*N *suppose we align Y to X), while allowing two conditions:

(1) Multiple mapping, *i.e*. one time point in one time series may correspond to many time points in the other time series, and vice versa.

(2) Balanced alignment. We require the same number of time points from the two time series, to be selected for alignment. That is, we will have exactly (*M*-*N*) omitted points, assuming *M*>*N*.

With the above two constraints, the goal is to find a pathway with least weight from the bottom row to the top row, with the nodes in the path covering exactly *N *columns. This additional information that covers exactly *N *columns cannot be kept in the subproblem since we will query the subproblem with less columns. Therefore, we define a warping matrix as Mat[*N*][*M*][*M*-*N*], and the element of Mat as Mat[i][j][k]. This is the best solution to align the first i points in Y to the first j points in X, and only (j-k) points in X will be aligned to the first i points in Y.

In the above procedure, for each cell, time complexity of *O*(*M*) is required. By introducing an indirect recursion and maintaining two matrices, we can decrease the time complexity from *O*(*M*) to *O*(1) for each cell, and express the time complexity for the algorithm as *O(M × N × (M-N))*. Assuming *N*, *M *are the same order, the time complexity for our algorithm is *O*(*M*^3). In comparison, if *O*(*M*) is required for each cell, the time complexity will be *O*(*M*^4). For example, running our DTW-S algorithm on 1,000 genes with *M *= 40 and *N *= 20, on a machine with a single core processor (1.83 GHz speed and 2 GB memory), takes only 12 seconds.

**Figure 1:**
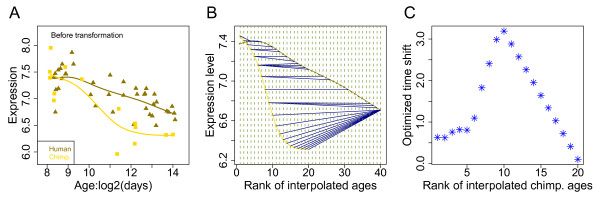
**Illustration of time series alignment using DTW-S**. (A) The x-axis shows human (dark yellow) and chimpanzee (yellow) age on a log2-scale, including conception time following [15]. The y-axis shows expression of an example gene (*VTA1*) on a log2-scale. The triangles and the diamonds represent real expression values. The curves represent the spline curves. (B) The alignment results for the DTW-S procedure. The x-axis represents the rank of ages for interpolated points. The circles on the curves represent the interpolated expression values. The blue lines show alignment matches between the two interpolated time series calculated by the DTW-S algorithm. (C) The optimized time shift estimates for chimpanzee ages based on the expression pattern of *VTA1*, after aligning the chimpanzee time series to the human time series. (D) The y-axis shows the expression level after shifting chimpanzee expression time series by the magnitude of the time shifts estimated by the DTW-S algorithm (shown in C).

We use an example gene expression profile (VTA1) of human and chimpanzee brain maturation to illustrate our procedure [15]. The dataset contains gene expression time series from 39 humans (0-47 years of age) and 14 chimpanzees (0-44 years of age). The ages of sampled individuals are distributed non-uniformly between birth and maturity (Additional file 1, Figure S1A). First, we estimate gene expression trajectories using splines (cubic polynomial interpolations with four degrees of freedom). Next, we interpolate gene expression values at 40 time points within human time series (X) and 20 time points within chimpanzee time series (Y), distributed uniformly over the sampled time intervals (*M *= 40, *N *= 20). An example of this procedure is illustrated in Figure 1A.

Next, we align interpolated values of the Y time series to the X time series using the dynamic programming-based algorithm as described above. As a result, among all possible alignment combinations that preserve the order of the time series, the one with the minimal Euclidean distance between *N *time points subset from a total of *M *time points of X time series, and *N *time points from Y time series, is selected as the optimal alignment between X and Y. An alignment example is shown in Figure 1B.

Based on this optimal alignment, we further calculate the time shift between the two time series as the difference between time of point within Y and the subset of X. Specifically, if Y_i _is aligned to X_j_, shift(Y_i_) = time(X_j_)-time(Y_i_). If Y_i _is aligned to (X_j_,..., X_j+q_), shift(Y_i_) = mean(time(X_j_),..., time(X_j+q_))- time(Y_i_). We illustrate the optimized shift for an example gene in Figure 1C.

Since our algorithm does not suit the analysis of genes that have substantially different temporal expression behavior in different time series (as such genes might involve factors other than change in expression timing), we recommend that users filter the tested genes by a correlation test.

The warping algorithm is written in C and R languages and is available within the R package *TimeShift *at http://www.picb.ac.cn/Comparative/data.html.

### Estimating the significance of time shifts and the false positive rate

A novel feature of our algorithm is that it calculates the significance of the time shift estimate per gene (with the null hypothesis of zero time shift) and determines the FPR. For this, we designed the following simulation procedure: suppose that we have *N_g _*genes, each of which contains two gene expression series x and y, with length m and n respectively. We apply the above-described DTW-S steps to these *N_g _*genes and calculate the time shift gene-by-gene for each time point. We call these estimates "real shifts". Then, for each gene, we construct a background zero-time-shift distribution. Specifically, we simulate two time series, based on one of the two original time series, with zero time shift between them and estimate "simulated shift" values 1,000 times. Next, we create 50 more simulated time series with zero time shift, and construct 1,000 background zero-time-shift simulations for each of them. Finally we compare differences between the real shift and the background zero-time-shift distribution, and 50 simulated zero-time-shift time series, to their background zero-time-shift distributions. We then estimate the final significance of the time shift, based on the difference between these two differences.

In detail, the procedure runs as follows:

1. For each gene, build a spline model called *Smodel *to fit one of the time series (either x or y) from the original dataset and calculate the residuals. Next, construct a model error distribution (*Nerror*) as a normal distribution, with the mean and the variance equal to the mean and the variance of the residuals.

2. Simulate two time series with zero shift by modeling the expression levels for the real time points of the x and y time series, using the same *Smodel *for both and adding errors based on the *Nerror *distribution. Repeat this step 1,000 times.

3. Apply DTW-S to the simulated time series to obtain shift estimates for each time point of each gene. For each time point, count the proportion of simulations with the absolute value of shift greater than or equal to the absolute value of real shift. Calculate a *p *-value for randomly observing a shift as large as that observed. Then, count the number of significant time shift points per gene (in the analysis we used the nominal cut-off *p *< 0.05 for choosing significant time shift points). We term the distribution of the number of significant time points across all tested genes, the "real" distribution.

4. For each gene, generate 50 extra pairs of simulated time series with zero shift following steps (1) and (2), and estimate the time shift by DTW-S. For each of these 50 zero-time-shift time series, compare the time shifts to 1,000 simulated shifts in the same way as comparing the real shift to 1,000 simulated shifts in step (3). So, for each of the 50 simulations, and for each time point of each gene, we can determine how frequently zero-time-shift time series yielded significant time shift estimates compared to the 1,000 simulated shift distributions (false positives). Note that these false positive estimates are based on time series simulated using real data parameters on a gene-by-gene basis. Finally, count the number of significant false positive time shift points per gene, for each of the 50 zero-time-shift time series, and build a corresponding distribution of the number of significant false positive points across genes, called a "null" distribution.

5. For each of the significant time shift points per gene, we calculate the FPR as the proportion of genes with an equal or greater number of significant time shift points per gene in the "null" distribution, compared to the "real" distribution. Based on this comparison, the minimal number of significant time shift points per gene can be estimated at the desired FPR level. A gene is classified as having a significant shift if it contains the number of significant time shift points equal to or greater than this minimal number.

### Preparation of simulated datasets with known time shift

1. We generated a time series x with length m from a given type of model (x = f(t)). We used three types of function: sine, linear and polynomial (described below). To each of the n time points, we added random error sampled from a normal distribution. This gave us a first expression time series with time *t*.

2. We generated a second expression time series y with the same length *n*, using the same model (*y = f(t)*), and the same error distribution, as the first time series. However, for this series we shifted the time *t *in the model calculation by a shift of known magnitude: time shift = Δ*_1_,...*, Δ*_n_*, where Δ denotes shifts at each time point, which can themselves be a function of *t*.

3. We applied DTW-S on x and y, by first interpolating *M *and *N *uniformly distributed time points across x and y, to generate X and Y respectively (with *M*>*N*). Then we estimated the time shift of aligning Y to X by applying DTW-S.

4. The entire procedure was repeated 1,000 times, starting from the simulation of x and y. We finally compared the obtained time shift estimates to the original set values.

## Results and Discussion

### Testing DTW-S using heterochrony simulations

To test the efficiency of time shift estimation by DTW-S, we first applied the algorithm to a number of simulated datasets with known heterochrony.

#### Sine function

**Figure 2:**
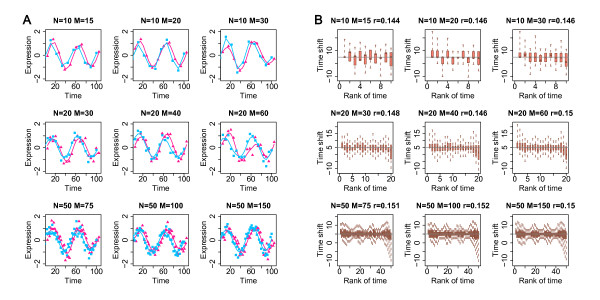
**Estimating constant time shift between two simulated time series**. (A) The data shown are expression levels of time series for species 1 (sp1, purple) and species 2 (sp2, light blue), modelled using the *sine *function with different sets of *N *and *M *parameters. We use the DTW-S procedure to align the sp2 time series to sp1, and calculate time shift values for the ages of sp2. The parameters *N *and *M *shown above the panels represent the numbers of interpolated time points for sp2 and sp1 time series, respectively. The number of the original time points *n = N*. (B) Time shift estimates calculated by the DTW-S procedure. Time shift estimates are based on 1,000 simulations of the expression time series shown in (A). The gray lines show the actual time shift values used in for data modelling. The parameter *r *represents the proportion of the total variance introduced by simulated error (ε).

We tested whether our method could be applied to periodic expression changes, such as expression changes associated with the cell cycle or circadian rhythm. Across the trials, we used the same error distribution parameters and time shift, while we varied the three parameters *n*, *N*, and *M *(these parameters reflect numbers of actual and interpolated time points, see Methods for Details). For simplicity, we assumed numbers of actual and interpolated time points to be equal (*n *= *N*). The expression changes with time were modeled as y = sin(π*25*/t)+ε*, with error *ε~N*(0,0.3) and fixed time shift Δ = 5 for all time points. Using these error distribution parameters, residual variance constituted approximately 15% of the total variance in the simulated datasets. This percentage of error-related variance is within the range of technical and biological variance, relative to factors such as age and species identity, observed in actual microarray experiments (*e.g*. [15]). These results demonstrate that DTW-S estimates are robust with respect to the numbers of original data points and interpolated time points. Furthermore, DTW-S could estimate the true time shift value (Figure 2). As expected, variation among time shift estimates decreased with increasing number of time points.

#### Linear and polynomial functions

We tested DTW-S on non-periodic gene expression changes, such as changes described by linear and polynomial curves. Most organismal processes, *e.g*. development, aging, or response to stress, involve such non-periodic patterns. Specifically, we tested the algorithm's ability to determine constant or variable time shifts, as well as it's performance at different levels of error variance (ε) and with a different sample size. We tested the algorithm using the following parameters: *n *= 20, *N *= 20 and *M *= 40.

First, we applied DTW-S on simulated time series based on linear and polynomial curves with a constant shift. We used the function *y *= *a*+*bt*+*dt*^2^+ε, with *a *= (0,1), *b *= (-1,0,1), *d *= (-1,0,1), to simulate expression time series. In addition, we used a fixed time shift Δ = 2 for all time points. We set the error distribution parameters at ~10% or ~20% of the total variance, *i.e*. 10% or 20% of the total variance is attributable to error. This proportion is within the error variance range observed in actual microarray experiments. Under both error rates and for both linear and polynomial curves, our method yielded accurate and robust time shift estimates (Additional file 1, Figures S2, S3).

**Figure 3:**
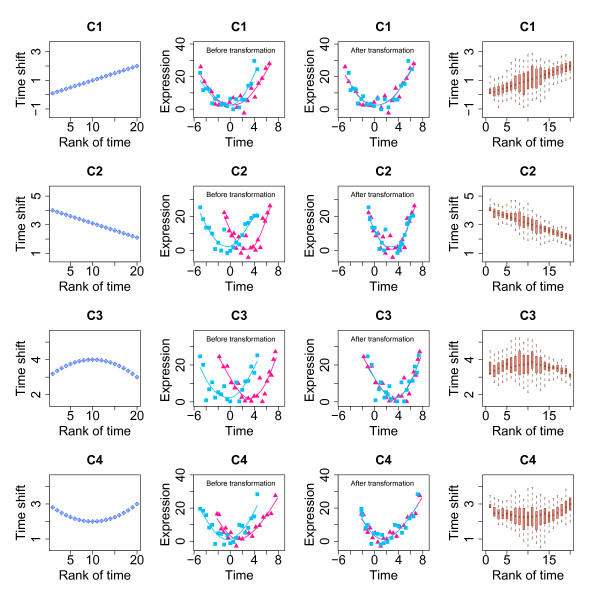
**Estimating simulated time shifts with DTW-S**. The leftmost columns show the distributions of the four sets of variable time shift (C1, C2, C3 and C4). In panels C1 and C2, the simulated time shift is increasing or decreasing linearly, while in panels C3 and C4, the time shift follows a quadratic trajectory. The second column from the left shows the corresponding two expression time series, sp1 (purple) and sp2 (light blue). These are simulated based on the function *y *= 1+*t*+*t*^2^+ε (*y *is the expression level, *t *is time and ε shows error), as well as on the time shifts following the patterns C1 to C4. The third column from the left shows the two expression time series after shifting sp2 to sp1 by the magnitude of the shifts estimated by the DTW-S. The rightmost columns display the corresponding time shift values estimated by the DTW-S algorithm in 1,000 simulations of sp1 and sp2 time series, with random error ε~*N*(0, 4).

Second, we tested the ability of our method to determine variable time shifts between two time series. In a biological system, it can be reasonably assumed that the difference in timing of expression changes between two processes might increase or decrease gradually. We therefore used four sets of variant time shift (the linear shifts: C1 and C2, and the polynomial shifts: C3 and C4) to simulate expression time series, two linear and two quadratic (*y *= 3+2*t*-*t*^2^+ε and *y *= 3-2*t*+*t*^2^+ε), with error ε~*Ν*(0,4), such that residual variance constituted ~20% of the total variance. Our results showed that under these conditions DTW-S can effectively identify the modeled variable time shift between two time series (Figure 3). Specifically, in simulations for non-linear shift patterns (C3 and C4 in Figure 3), the predicted time shifts were significantly better described by the original polynomial shift models, than they were by linear models (ANOVA *p *< 0.01).

Next, we assigned the same four sets of variant time shift (C1, C2, C3, and C4) to time series, modeled using combinations of the expression profile parameters *a*, *b*, *d *(*i.e*. linear and polynomial curves), with ~20% residual variance. We found that, irrespective of the expression curve type and the time shift type, our method robustly identified the original shift profile between time series (Additional file 1, Figure S4).

Lastly, we tested our method's performance under 30% residual variance. Overall, DTW-S performed well at this relatively high error rate. Compared to the original time shift, the estimated time shifts showed higher variance at the middle of the age distribution, and biases towards positive and negative time shift estimates at the beginning and end of the age distribution respectively (Additional file 1, Figure S5). We note that, under even higher levels of noise, DTW-S might not be able to distinguish a non-linear time shift trajectory from a linear one. In these cases, it might still be possible to accurately estimate the magnitude and type of shift by averaging across multiple genes to minimize noise.

### Application to primate brain development

#### Human and chimpanzee cortical development

**Figure 4:**
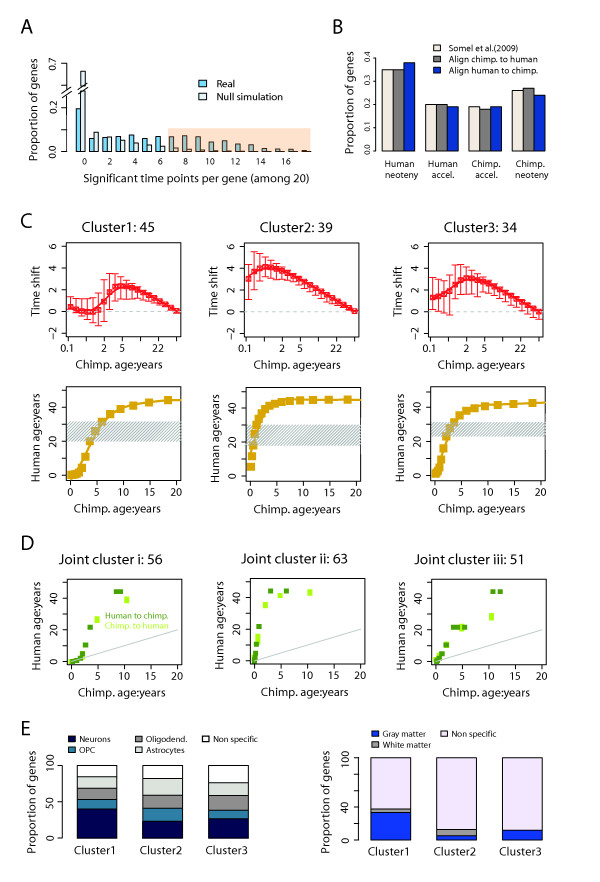
**Human-chimpanzee expression heterochrony analyzed by DTW-S**. (A) Numbers of significant time shift points per gene for 1183 genes tested for heterochrony in the primate brain expression dataset. The x-axis shows the number of interpolated time points (out of a total of 20) with significant time shift (*p *< 0.05) estimated using DTW-S (blue), as well as the mean time points in 50 simulated datasets (light blue). The simulation is based on the human expression time series (see Methods). In this case, the FPR is ~10% for genes with ≥7 points showing significant time shift (denoted by the light pink shaded area). In this analysis, we have aligned chimpanzee expression time series to human time series. (B) Proportions of genes assigned to four phylo-ontogenetic categories. The labels are the four phylo-ontogenetic categories (see Results). The colors indicate gene classification into the phylo-ontogenetic categories stated in the original publication [15], carried out using the DTW-S algorithm by aligning chimpanzee expression time series to human time series, or aligning human expression time series to chimpanzee time series. (C) Patterns of time shift between human and chimpanzee time series for genes with human-specific neotenic expression, calculated by aligning chimpanzee expression time series to human time series. The numbers above each graph represent numbers of genes within the cluster. The upper row panels show time shift estimates for mean shift based on all genes in a cluster (red lines) and the distribution of the shifts (arrows) for individual genes within clusters (5%-95%). Positive values of time shift indicate that human ages are mapped to younger ages in the chimpanzee time series, indicating slower/delayed human development. Time shift shown on the y-axis is calculated as the base-two logarithm ratio between chimpanzee age and human age at aligned expression time points. The bottom row panels show the relationship between human and chimpanzee ages, based on the calculated time shift estimates shown on the upper row (yellow lines). Shaded areas show the human age range corresponding to the maximum shift interval. (D) Panels show the relationship between human and chimpanzee ages, based on all the calculated significant time shifts between human and chimpanzee time series for genes with human-specific neotenic expression. The genes are identified as neotenic either by aligning chimpanzee to human time series, or by aligning human to chimpanzee time series. The numbers above each graph represent numbers of genes within the cluster. (E) Properties of genes in three clusters defined based on their human-chimpanzee time shift patterns as shown in (C). The left panel shows the proportion of genes in each of the three clusters that overlap with genes that are expressed preferentially in a particular cell type: neurons, oligodendrocyte precursor cells (OPC), oligodendrocytes, astrocytes or non-specific. The right panel shows the proportions of genes in each of the three clusters that overlap with genes that are expressed preferentially in gray matter, white matter or non-specific.

We first applied the DTW-S algorithm to investigate the heterochrony patterns between the brain developmental profiles of humans and chimpanzees. Prevalence of a specific type of heterochrony, delayed development in humans (or neoteny), was previously reported in human and chimpanzee brains [15]. This result was based on expression time series measured in the dorsolateral prefrontal cortex in 39 humans (0-47 years), 14 chimpanzees (0-44 years) and one outgroup species: 9 rhesus macaques (1-18 years). For all three species, the dataset covered most of the postnatal ontogenesis and maturation, from birth until adulthood (Additional file 1, Figure S1A). In this study, we re-examined this work using DTW-S. Our hypothesis was that expression heterochrony patterns might vary among genes, even among those that show the same type of heterochrony, with regard to the timing of the shift, as well as shift magnitude. Following the original analysis, we first selected 1183 genes satisfying the following criteria: (1) significant expression changes with age (using polynomial regression models, at F-test *p *< 0.05), (2) significant expression difference between humans and chimpanzees (using analysis of covariance (ANCOVA), at F-test *p *< 0.05) and, (3) significant positive correlation between human and chimpanzee expression profiles (Pearson correlation *p *< 0.05 and *r *> 0). We then applied the DTW-S algorithm to these 1183 genes to identify expression trajectories with significant time shift between the two species. Here, we aligned the chimpanzee time series to the human age-scale. For each gene, we set the *p*-value cutoff for the significant time shift points at 5%, and the gene significance cutoff as more than one third of the gene's time points having significant time shift (Methods). At these cutoffs, 482 out of the 1183 genes tested had significant time shift, and the FPR was estimated at 10.7% (Figure 4A).

We assigned these genes to one of four phylo-ontogenetic categories, using our previously reported classifications [15]: (a) human-specific neotenic expression (here neoteny denotes a slow-down or delay in development), (b) human-specific accelerated expression, (c) chimpanzee-specific neotenic expression and, (d) chimpanzee-specific accelerated expression (Additional file 1, Figure S6). To do so, we first determined the direction of the time shift (acceleration, neoteny, or none) between the human and chimpanzee time series, for all 482 genes with significant time shift. If at least 70% of time points showed consistent shift direction, we classified the related gene as accelerated or neotenic. We then determined whether expression change has happened to the human or to the chimpanzee evolutionary lineage, using the rhesus macaque time series as the outgroup (Methods). For all 482 genes, we could assign 337 (at lineage assignment test *p *< 0.05), or 267 (at lineage assignment test *p *< 0.01), to one of the four phylo-ontogenetic categories. This analysis yielded a clear excess of genes in the human-specific neotenic expression category (Figure 4B), which adds support to our previously published results [15].

To test the robustness of our method, we reversed the entire time shift identification procedure by aligning the human time series to the chimpanzee trajectory. This yielded 642 genes with significant shift (FPR = 8.7%, *p*-value < 0.05, number of significant time shift points per gene = 7). The difference in the numbers of genes with significant time shift compared to the previous procedure, is most likely related to sample size differences between the chimpanzee and the human time series (Methods). Despite this variation, the two sets of genes identified in the two procedures largely overlap: 369 between 482 and 642 genes (Fisher exact test: *p *< 0.0001). The proportions of genes assigned to the four categories were also highly consistent (Figure 4B).

In the previous study, any differences in time shift magnitude among the heterochronic genes, were ignored. Furthermore, the time shift was assumed to remain constant across all time points [15]. To test these assumptions, we analyzed the time shift patterns estimated by DTW-S for 118 human-specific neotenic genes. Using hierarchical clustering we found that, instead of forming a single pattern, the time shift profiles of these genes fell into three distinct time shift clusters (Additional file 1, Figure S7). Clustering time shift profiles using the k-means algorithm also produced consistent segregation into three clusters. In 828 out of 1,000 iterations, the k-means algorithm yielded the same distribution of time shift patterns across the three clusters. The three clusters also showed marked differences in both the timing and the amplitude of the time shift (Figure 4C). Specifically, all three clusters showed time shift peaks at 20-30 years of age in humans, but at different ages in the chimpanzees: 1 year (Cluster2), 2-4 years (Cluster3), and 4-6 years (Cluster1) (Figure 4C).

To test the robustness of the time shift estimates for the three clusters, we combined the estimates obtained in the chimpanzee-to-human alignment, and in the human-to-chimpanzee alignment, and repeated the clustering using the k-means algorithm. This approach also led to robust segregation into three clusters. In addition, for the 170 genes with significant time shift estimates in at least one of the two alignments, both direct and reverse alignments produced consistent time shift estimates between human and chimpanzee expression profiles within each of the three clusters (Figure 4D).

Finally, we investigated whether the various patterns of heterochrony identified by our approach may be associated with specific biological functions. We first tested the characteristics of genes in the three time shift clusters using published data for gene expression specific to human brain gray and white matter [21], as well as on cell type-specific expression in the mouse central nervous system [22]. Genes in Cluster1 were significantly enriched among genes preferentially expressed in human gray matter (hypergeometric test [HT] *p *= 0.001), while genes in the other two clusters did not show any cell type specificity ([HT] *p *> 0.5) (Figure 4E). Consistently, genes in Cluster1, but not in the other two clusters, tended to be enriched among neuron-specific genes ([HT] *p *= 0.058) (Figure 4E). These results indicate that differences in time shift patterns, identified by DTW-S, might reflect differences in ontogenetic timing of gene expression changes among different human brain cell types, and between histological locations.

#### Development of the human and macaque prefrontal cortex and cerebellum

To further test the utility of the DTW-S in uncovering novel biological features, we studied time shift patterns in human and rhesus macaque brain development in two distinct regions: prefrontal cortex and cerebellum. For this, we used a published expression dataset containing time series measured in the dorsolateral prefrontal cortexof 23 humans and 26 rhesus macaques, and in the cerebellum of 22 humans and 24 rhesus macaques [23]. In both species, the dataset covered most of the lifespan, humans: 0-98 years, macaques: 0-28 years (Additional file 1, Figure S1B).

Based on these data, we selected 1084 genes in the cortex and 950 genes in the cerebellum that satisfied the following criteria: (1) significant expression changes with age (using polynomial regression models, at F-test *p *< 0.05), (2) significant expression differences between humans and rhesus macaques (using analysis of covariance (ANCOVA), at F-test *p *< 0.05), (3) significant positive correlation between human and rhesus macaque expression profiles (Pearson correlation *p *< 0.05 and *r *> 0) and, (4) significant time shift between human and rhesus macaque expression profiles (FPR = 11.7% for cortex and 11.5% for cerebellum). Taking the union of the genes that passed these criteria in the cortex or cerebellum yielded 1735 genes.

For these genes, we determined the direction of the time shift (acceleration, neoteny, or none) between human and rhesus macaque time series, in both cortex and cerebellum, by aligning rhesus macaque data to the human age scale. If at least 70% of time points showed consistent time shift direction, we classified this gene as accelerated or neotenic. Following this procedure, the vast majority of genes were classified as neotenic, with 1444 (83%) and 1469 (85%) in the cortex and cerebellum respectively. This result is consistent with the faster rate of macaque brain development and maturation that has been previously reported [24].

**Figure 5:**
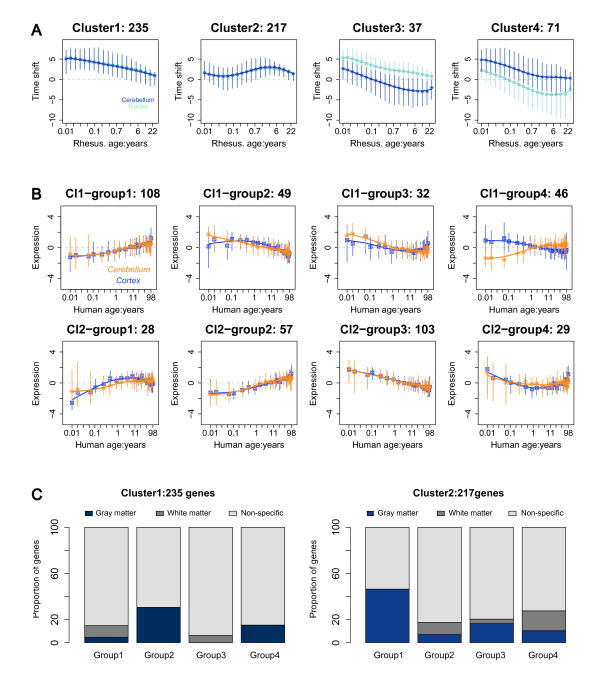
**Human-rhesus macaque time shift analyzed by DTW-S**. (A) Four patterns of time shift between human and rhesus macaque expression time series, showing high time shift profile correlation (*r*: 0.5~1) between cortex and cerebellum. The time shift was calculated by aligning rhesus expression time series to human time series. The numbers above the graphs represent numbers of genes within each cluster. The graphs show mean time shift of all genes within a cluster (points) and the time shift interval for individual genes within a cluster (5%-95%). Positive values of time shift indicate that human ages are mapped to younger ages in the rhesus macaque time series, indicating slower/delayed human development. Time shift shown on the y-axis is calculated as the base-two logarithm ratio between rhesus macaque age and human age at aligned expression time points. (B) Patterns of expression profiles of human genes in the time shift Cluster1 and Cluster2 shown in (A). The numbers above each graph represent genes within a group. The graphs show mean expression of all genes within a group (points) and expression level variance (error bars) for individual genes within a cluster (5%-95%). (C) Properties of genes in the time shift Cluster1 and Cluster2. The left and the right panels show the proportions of genes in each of the four groups of Cluster1, and Cluster2 respectively, that overlap with genes with expression specific to gray matter, white matter or non-specific.

Overall, time shift measurements correlated positively between the prefrontal cortex and cerebellum (Additional file 1, Figure S8), with 560 out of 1750 genes tested showed strong positive correlation (Pearson *p *< 0.05, *r *> 0.5). The time shift profiles of these genes could be assigned to four clusters using the k-means clustering method (Figure 5A). Notably, the vast majority of these genes (452 genes in clusters 1 and 2) showed nearly identical time shift profiles in the prefrontal cortex and cerebellum. Does this similarity of time shift profiles reflect similarity in gene expression profiles in the two brain regions? Indeed, for some genes in both clusters 1 and 2, gene expression profiles in cortex and cerebellum followed the same trajectories (Figure 5B). Other genes, however, showed clearly distinct expression profiles in the two brain regions, while still sharing the same time shift profile. In a search for a biological explanation of this phenomenon, we tested cell type specificity and enrichment in biological functions specified by Gene Ontology (GO) annotation [25]. We found that genes grouped into Cluster1 based on their time shift profiles, and showing identical expression patterns in cortex and cerebellum (Cl1-group1), were enriched in white matter ([HT] *p *= 0.0002), and were annotated in GO terms: "lipid metabolic process" ([HT] *p *= 0.007) and "cellular lipid metabolic process" ([HT] *p *= 0.007). By contrast, Cluster1 genes showing different expression patterns in cortex and cerebellum (Cl1-group2) were enriched in gray matter ([HT] *p *= 0.0004) and annotated by GO as "nervous system development" ([HT] *p *= 0.05) (Figure 5C). Similarly, genes grouped into Cluster2 based on their time shift profiles, and showing identical expression patterns in cortex and cerebellum (Cl2-group2), were enriched in white matter ([HT] *p *= 0.02) and mature oligodendrocytes ([HT] *p *= 0.03), while genes showing different expression profiles (Cl2-group1) were enriched in gray matter ([HT] *p *= 0.006). Thus, our results revealed an interesting biological phenomenon: within one species, ontogenetic profiles are shared between the prefrontal cortex and cerebellum for genes expressed in white matter, but distinct for genes expressed in gray matter. Importantly, however, the time shift between the human and rhesus macaque ontogenetic profiles is perfectly synchronized for both white and gray matter genes. On an organismal level this observation might not be surprising. Changes in the rate of ontogenesis might be expected to operate on the brain as a whole, leading to synchronized delay in white and gray matter development in humans, compared to rhesus macaques. Our results confirm that, on the gene expression level, such synchronization can indeed be observed.

## Conclusions

Comparisons of developmental patterns across closely related species are playing an increasingly important role in extracting meaningful information from biological time series. By modifying the dynamic time warping algorithm (DTW), we have designed an effective tool for time series alignment (DTW-S). Our simulation results show that this method is effective in calculating time shift between two time series, even when the proportion of noise is 20-30% of the total variance. Furthermore, this method performs well for expression profiles containing both recurrent and non-recurrent changes, and can estimate variation in the amplitude and direction of the time shift.

When we applied our method to a published gene expression dataset of human, chimpanzee and rhesus macaque brain development and maturation, we obtained robust and reproducible time shift estimates consistent with previous observations [15]. Furthermore, our method allowed us to classify genes into distinct categories according to their time shift patterns. This provided additional insights into biological mechanisms underlying human-specific brain development and maturation, which could not be deduced from the gene expression profile data alone.

Applying our method to a gene expression dataset of human and rhesus macaque brain development and aging, we found that genes showing synchronized time shift between the species, in the prefrontal cortex and cerebellum, do not always follow the same expression profiles in the two brain regions. Notably, genes showing both synchronized time shift between human and macaque ontogenetic trajectories, and synchronized expression patterns in the prefrontal cortex and cerebellum, were preferentially expressed in brain white matter. By contrast, genes showing synchronized time shift between human and macaque ontogenetic trajectories, but different expression patterns in the prefrontal cortex and cerebellum, were preferentially expressed in brain gray matter.

Taken together, these two examples demonstrate that the combination of gene expression time series profiles with ontogenetic time shift estimates provides additional information revealing the biological properties of the investigated system. The development of DTW-S algorithm, freely available as the R package "*TimeShift*", should facilitate the application of this approach to further studies.

## Authors' contributions

YY came up with the general idea and developed the algorithm and applications; SN wrote the code for the DTW-S algorithm; AGHX developed the R package; YPPC, MV, MS and PK supervised the analyses; LT provided helpful suggestions; YY, MS and PK wrote the manuscript. All authors read and approved the final manuscript.

## Authors' Information

YPPC, MV, MS and PK jointly supervised this study.

## Supplementary Material

Additional file 1 **Supplementary figures**. includes all additional figures mentioned in this paper. Click here for file
