# Supplementary material for: Development and application of a modified dynamic time warping algorithm (DTW-S) to analyses of primate brain expression time series
Source: BMC Bioinformatics. 2011 Aug 18;12:347. doi: 10.1186/1471-2105-12-347 (PMC3180390; doi:10.1186/1471-2105-12-347)
Supplement: Additional file 1 — Supplementary figures. includes all additional figures mentioned in this paper. [file 1471-2105-12-347-S1.DOCX]

Development and application of a modified dynamic time warping algorithm (DTW-S) to analyses of primate brain expression time series

**Supplementary Figures**


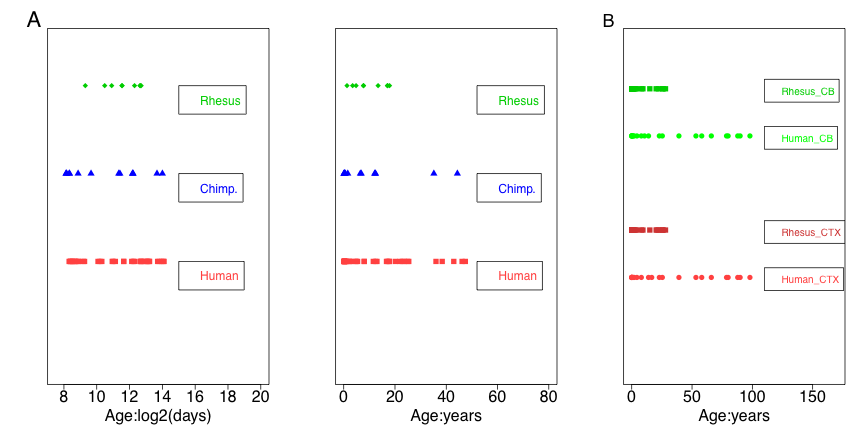


**Figure S1. Ages for all primate samples and human samples.** (A) X-axis shows the real ages, including conception time, of all samples from human, chimpanzee and rhesus macaque, measured on a log2-age scale and real age scale. (B) X-axis shows the real ages of all samples from human and rhesus macaque cortex and cerebellum, measured on a real-age scale.

A


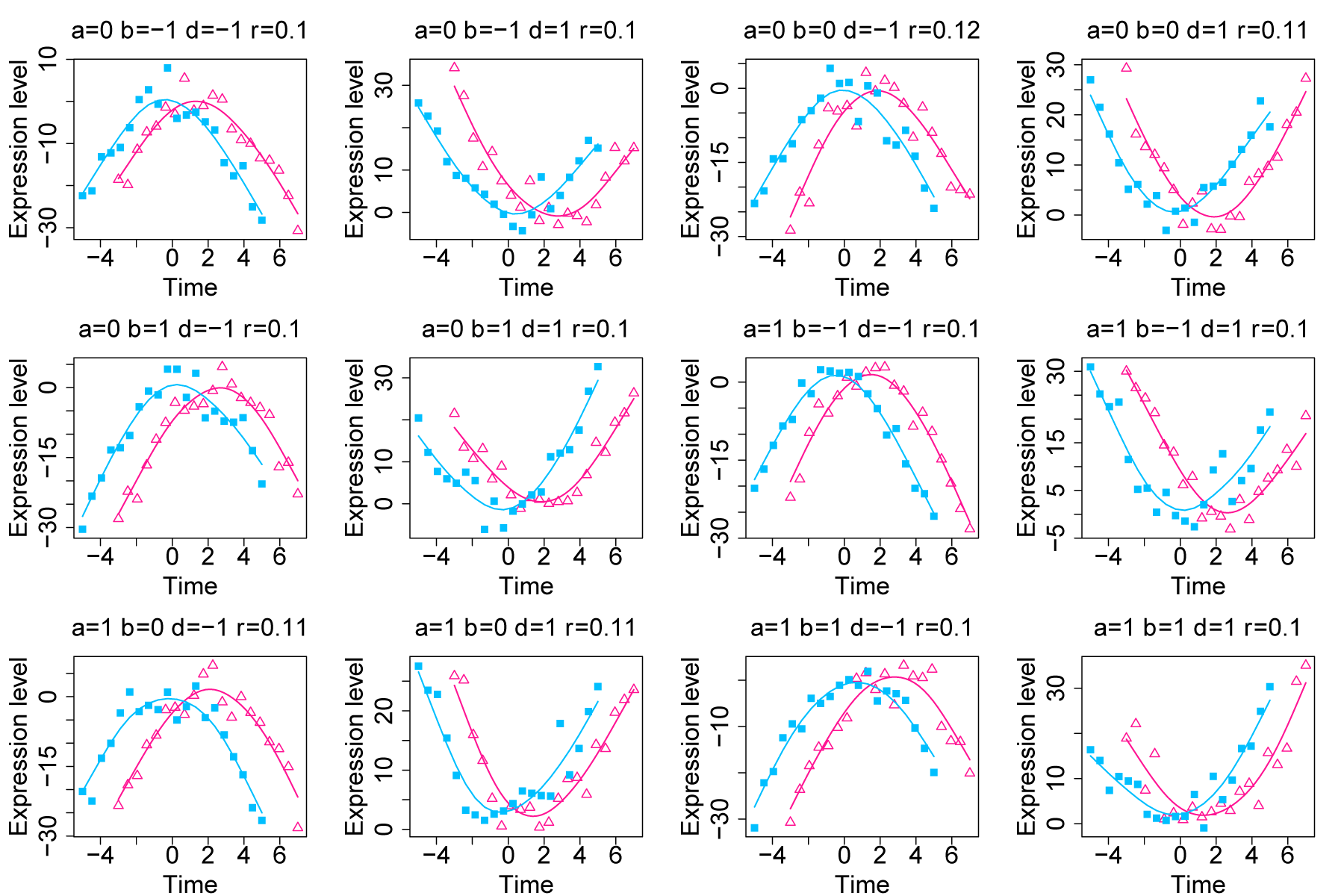


B


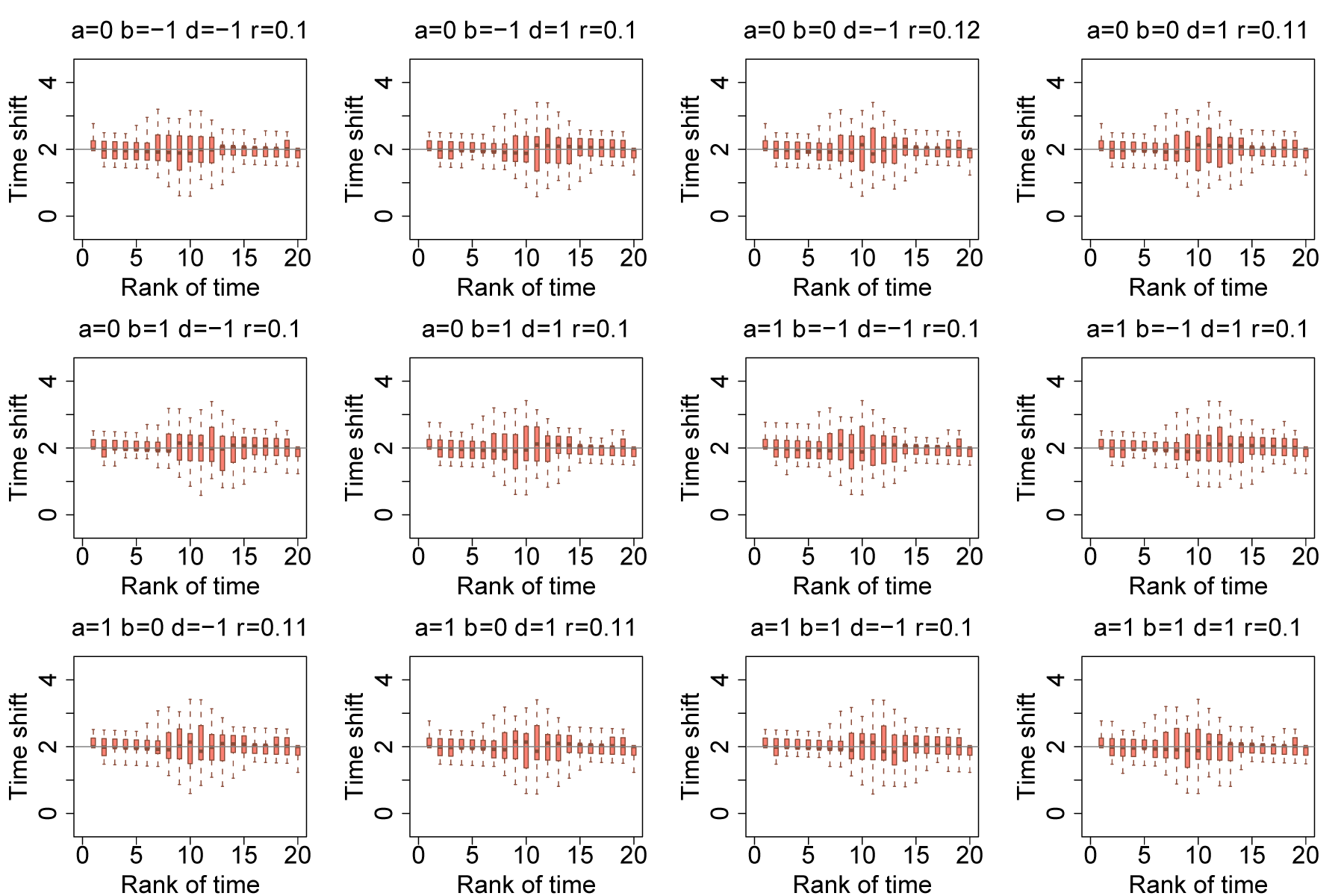


**Figure S2.** **Estimating constant time shift between two simulated nonlinear time series** (A) The panels show the expression profiles for two simulated time series, species 1 (sp1, purple) and species 2(sp2, light blue), modeled using function *y*=*a*+*bt*+*dt*^2^+*ε,* with the parameter values stated above each panel, and with random error *ε~N*(0, 3). The parameter *r* represents the proportion of the total variance introduced by simulated error (*ε*). We use the DTW-S procedure to align sp2 time series to sp1, and calculate time shift values for the ages of sp2 (modeled time shift=2 for all time points). (B) Time shift estimates for the time series shown in (A). The boxplots show the distributions of time shift estimates based on 1,000 simulations of expression for the time series shown in (A), with parameter values stated above each panel. The gray line shows the modeled time shift values.

A


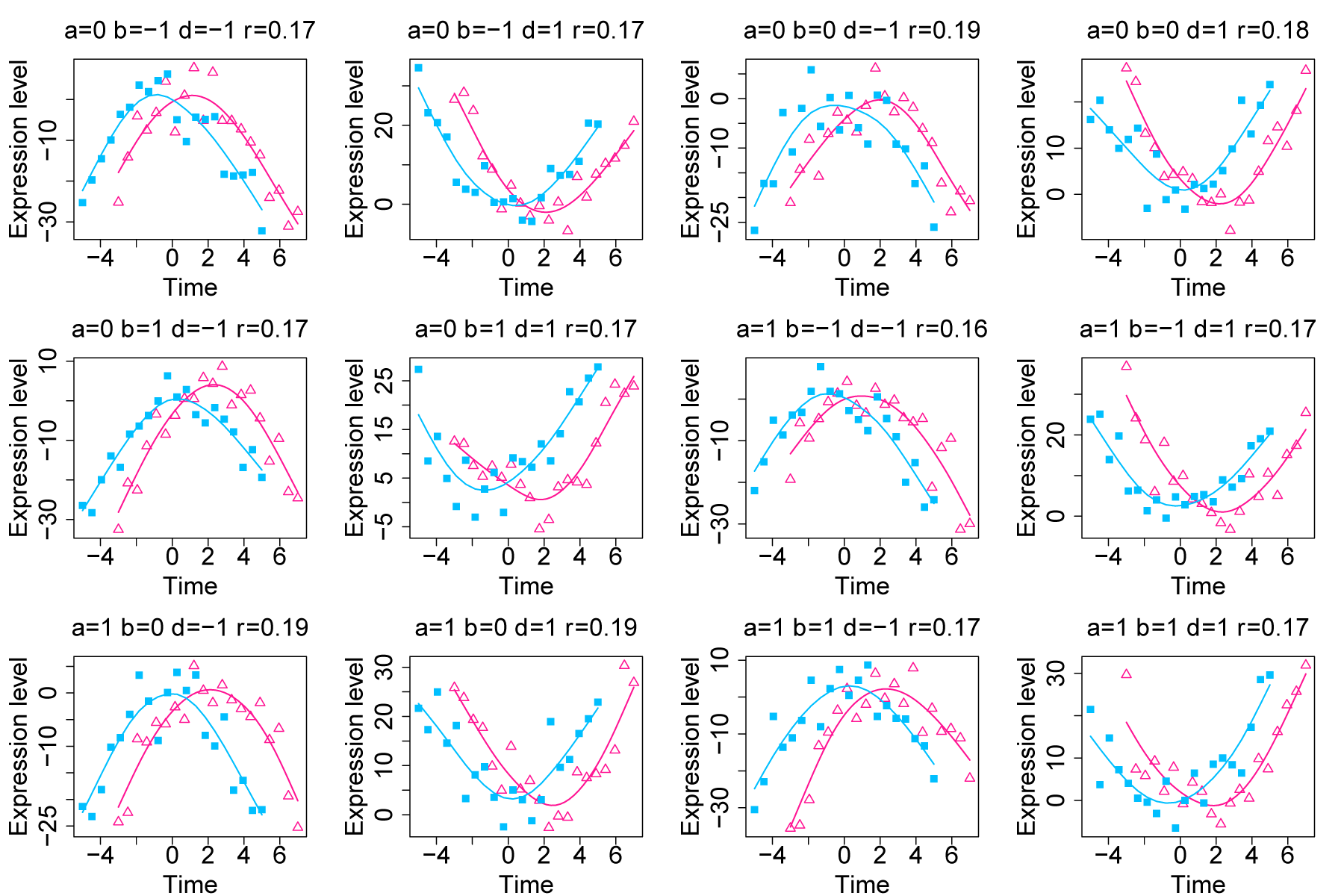


B


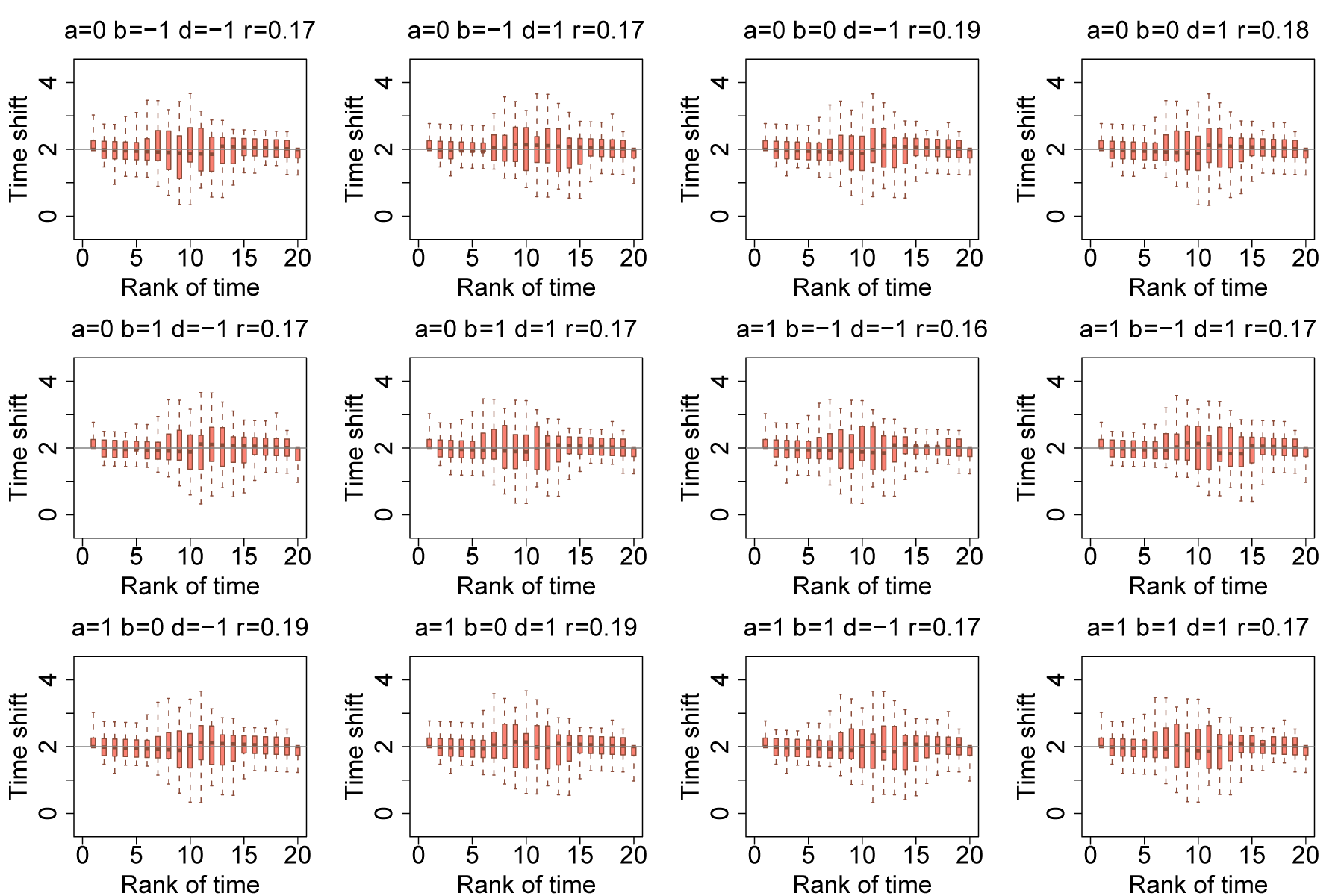


**Figure S3. Estimating constant time shift between two simulated nonlinear time series** (A) The panels show the expression profiles for two simulated time series, sp1 (purple) and sp2 (light blue), modeled using function *y*=*a*+*bt*+*dt*^2^+*ε,* with the parameter values shown above each panel, and with random error *ε~N*(0, 4). The parameter *r* represents the proportion of the total variance introduced by the simulated error (*ε*). We use the DTW-S procedure to align sp2 time series to sp1 and calculate time shift values for the ages of sp2 (modeled time shift=2 for all time points). (B) Time shifts estimates for the time series shown in (A). The boxplots show the distributions of time shifts estimates based on 1,000 simulations of expression for the time series shown in (A), with parameter values stated above each panel. The gray line shows the modeled time shift values.


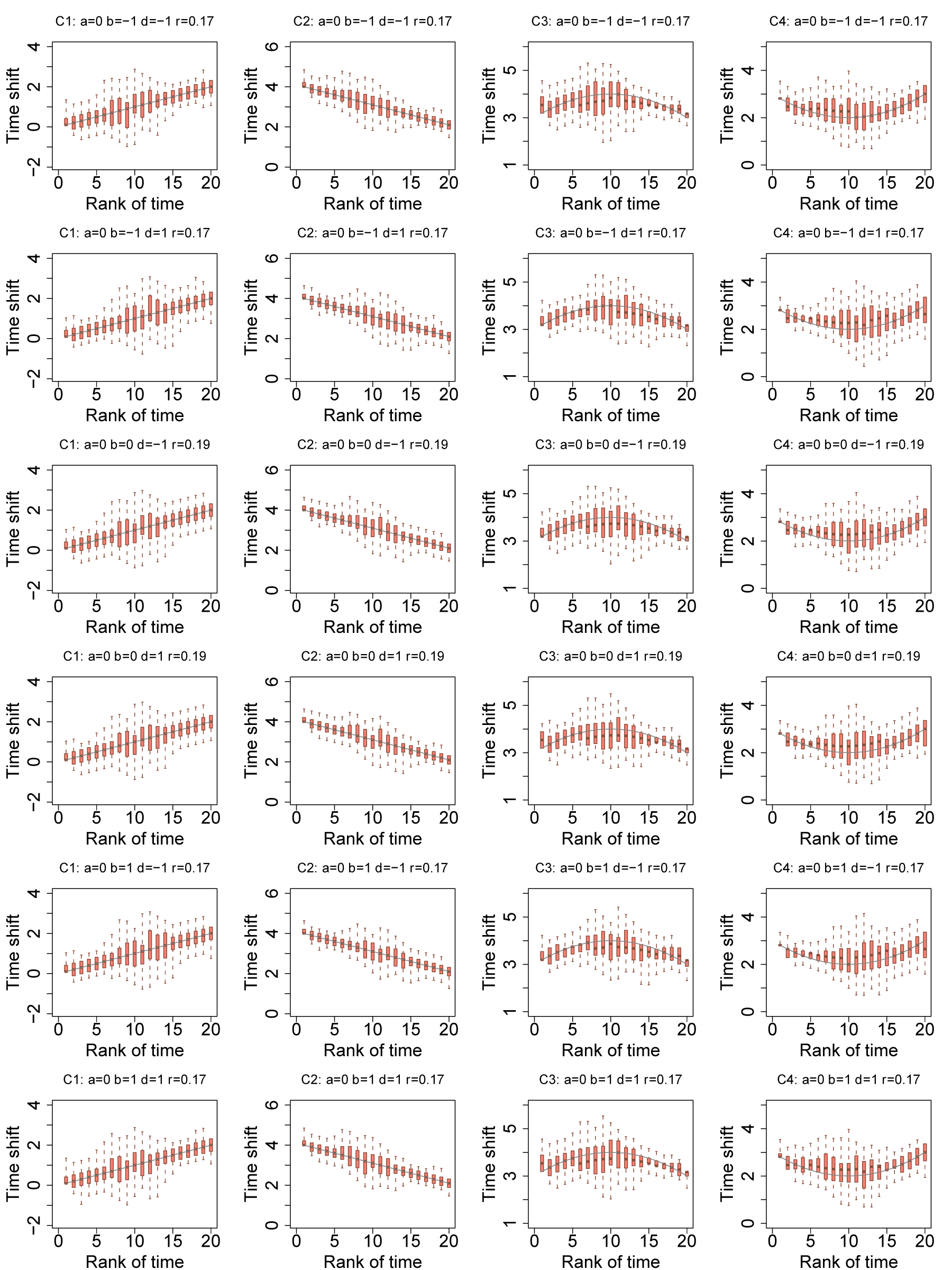


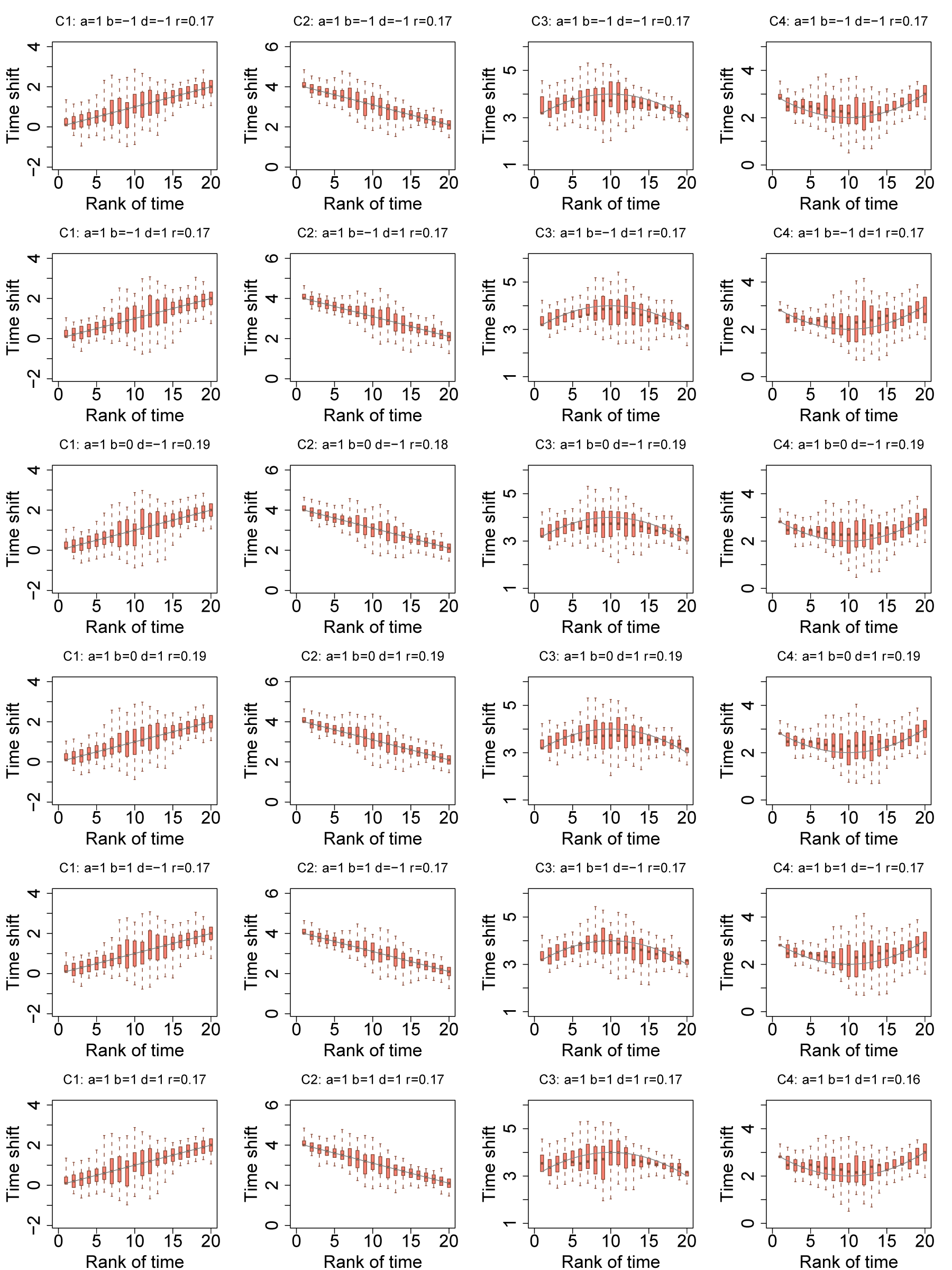


**Figure S4. Estimating variable time shift between simulated non-linear time series, with added random error accounting for ~20% of the total variance, using the DTW-S procedure**. The boxplots show the distributions of time shift estimates based on 1,000 simulations of expression of time series modeled using function *y*=*a*+*bt*+*dt*^2^+*ε*, with parameter values shown above each panel, and with random error *ε~N*(0, 4). The parameter *r* represents the proportion of the total variance introduced by simulated error (*ε*). The gray line shows the modeled time shift values that give the patterns C1, C2, C3, and C4 shown in Fig 3.


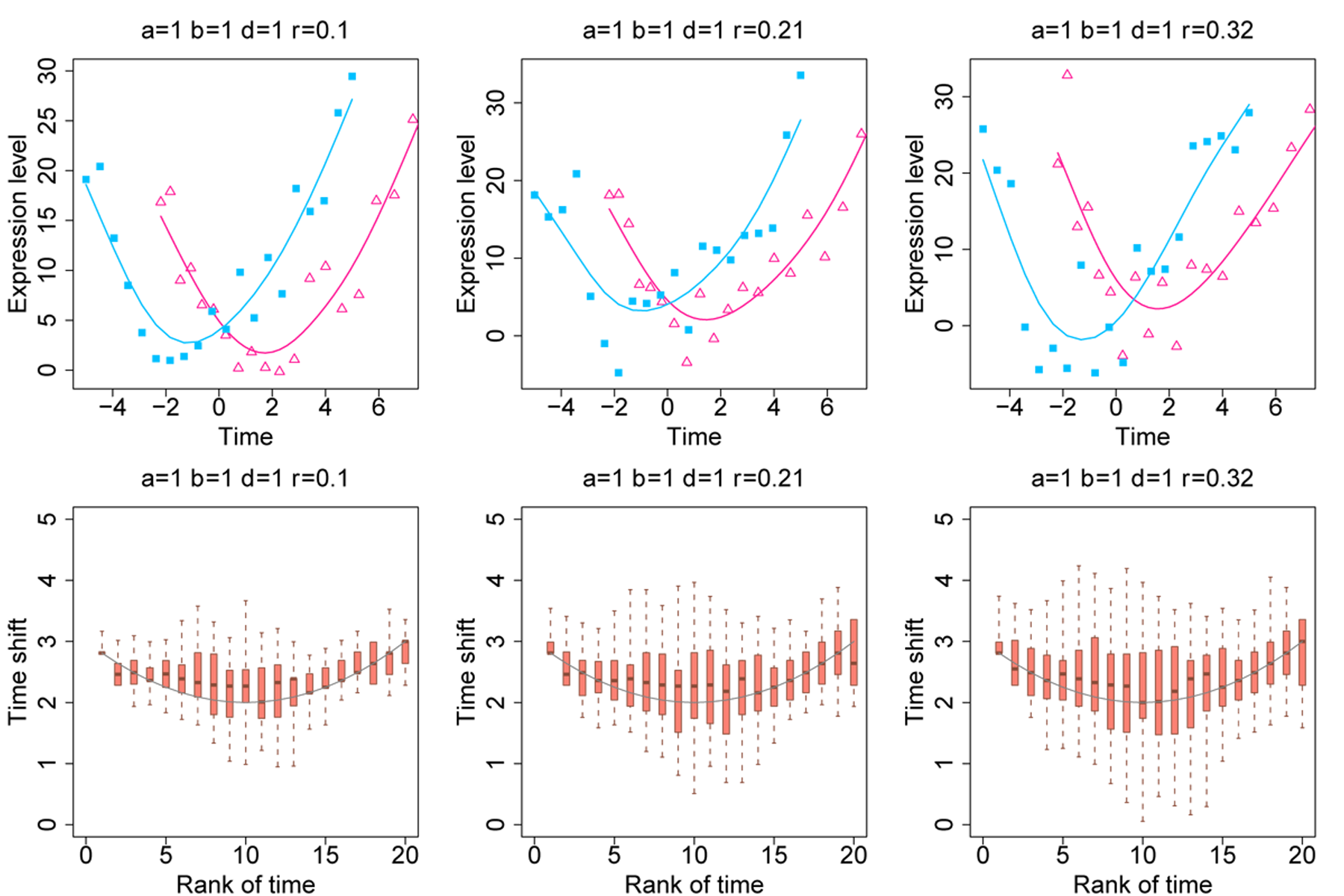


**Figure S5. Estimating variable time shift between simulated non-linear time series, with increasing proportion of added random error, using the DTW-S procedure.** The upper row panels show the expression profiles for two simulated time series, sp1 (purple) and sp2 (light blue), modeled using function *y*=*a*+*bt*+*dt*^2^+*ε,* with parameter values shown above each panel, and with random error: from left to right *ε~N*(0, 3), *N*(0,4.5) and *N*(0,6). The parameter *r* represents the proportion of the total variance introduced by simulated error (*ε*). We use the DTW-S procedure to align sp2 time series to sp1 and calculate time shift values for the ages of sp2. The boxplots in the bottom row panels show the distribution of time shift estimates based on 1,000 simulations of expression in the time series shown in the upper row. The gray line shows the modeled time shift values following the pattern of C4, shown in Fig 3.


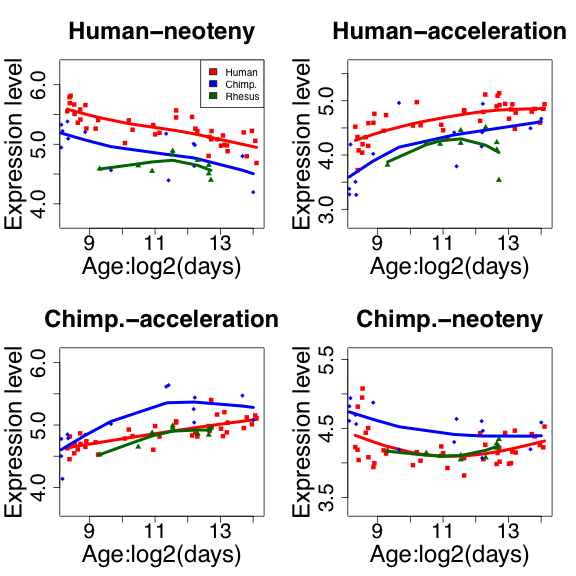


**Figure S6. Examples of gene expression patterns representing four phylo-ontogenetic categories.** The y-axis shows expression levels of four genes (clockwise from the upper left panel: TMEM132A, SKAP2, ST7 and KRIT1). The x-axis shows age in days plotted on a log2 scale. The colors represent species: red– human, blue–chimpanzee, green–rhesus macaque. Each dot represents an individual. The curves are fitted using a smoothing spline model with four degrees of freedom.


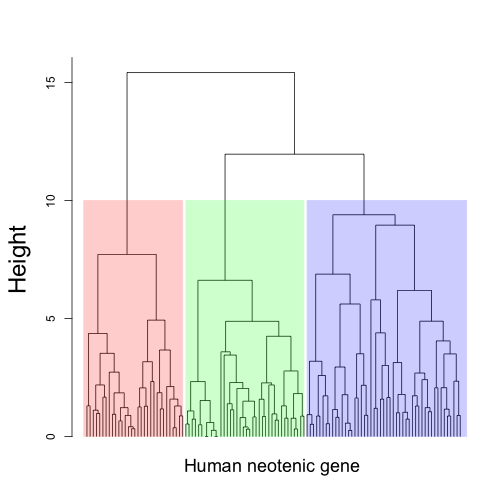


**Figure S7.** **Hierarchical clustering of time shift profiles.** The time shifts were calculated by aligning chimpanzee expression time series to human expression time series using the DTW-S procedure. The clustering analysis was carried out on 118 genes with human-specific neotenic expression patterns and significant time-shift estimates.


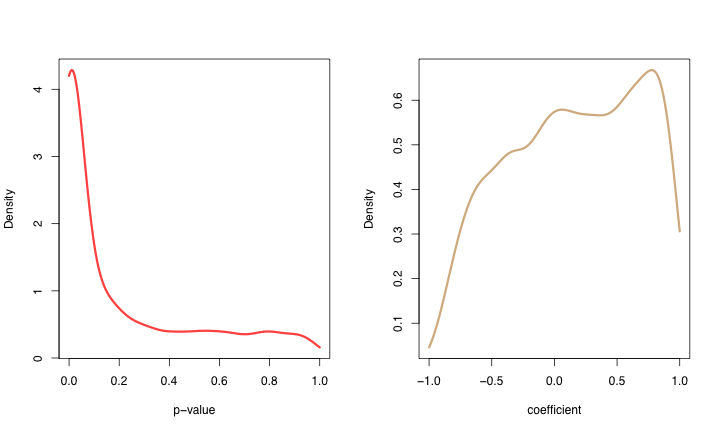


**Figure S8.** **Correlation between time shifts in human cortex and cerebellum for 1735 genes.** The left panel shows the *p-value* distribution of the Pearson correlation of the time shifts in the cortex and cerebellum. The right panel shows the correlation coefficients distribution.
